# Melatonin Signaling a Key Regulator of Glucose Homeostasis and Energy Metabolism

**DOI:** 10.3389/fendo.2019.00488

**Published:** 2019-07-17

**Authors:** Sharon Owino, Daniella D. C. Buonfiglio, Cynthia Tchio, Gianluca Tosini

**Affiliations:** ^1^Department of Pharmacology and Toxicology Morehouse School of Medicine, Neuroscience Institute, Atlanta, GA, United States; ^2^Department of Pharmacology, Emory University School of Medicine, Atlanta, GA, United States; ^3^Department of Physiology and Biophysics, Institute of Biomedical Sciences-I, University of São Paulo (USP), São Paulo, Brazil

**Keywords:** melatonin, MT_1_, MT_2_, diabetes, leptin

## Abstract

Melatonin, a hormone synthesized by both the pineal gland and retina, functions as an important modulator of a number of physiological functions. In addition to its rather well-established roles in the regulation of circadian rhythms, sleep, and reproduction, melatonin has also been identified as an important regulator of glucose metabolism. Recent genomic studies have also shown that disruption of melatonin receptors signaling may contribute to the pathogenesis of type 2 diabetes, although the exact mechanisms underlying its action remain unclear. Additionally, a large number of animal studies have highlighted a role for melatonin in the regulation of both glucose metabolism and energy balance. This review summarizes the current knowledge on the role that melatonin and its associated receptors play in the regulation of metabolism.

## Introduction

Melatonin is a hormone predominantly secreted by the pineal of vertebrates. The synthesis of melatonin occurs during the night via a rather straightforward biosynthetic pathway that involves four different enzymes. In many vertebrates (e.g., fishes, amphibians, reptiles, and birds) pineal melatonin synthesis is directly controlled by the circadian clock located in the pinealocytes, whereas in the mammalian pineal gland the synthesis of melatonin is controlled by a circadian clock located in the suprachiasmatic nucleus (SCN) of the hypothalamus. Due to its lipophilic nature, melatonin is not stored within the pineal gland, but rather diffuses into the blood and cerebral spinal fluid where its levels in these fluids reflects its synthesis (1).

Melatonin acts on many different cell types within the body where it exerts its effects as an antioxidant and free radical scavenger or functions via its G-protein coupled receptors named melatonin receptor 1 (MT_1_) and melatonin receptor 2 (MT_2_) (2). MT_1_ and MT_2_ are expressed throughout the body where they regulate the entrainment of circadian rhythms, sleep, blood pressure, and reproductive functions. Since these effects of melatonin have been reviewed by other authors, in both this issue of the journal, as well as by recent reviews in other journals, we have decided to focus our review on the role that melatonin plays in the regulation metabolism. Additionally, in the last section of our review, we also summarize how modern genetic studies that have implicated melatonin receptor polymorphisms in the regulation of a number of different pathologies.

## Melatonin as a Regulator of Metabolism and Body Weight

Within recent decades, a large number of animal studies using both pinealectomized rats and melatonin receptor knock out (KO) mice have begun to establish a rather unexpected role for melatonin in the regulation of glucose metabolism and energy balance. Early pinealectomy studies demonstrated that abolishing melatonin levels produces glucose intolerance and insulin resistance (3, 4). Interestingly, reintroducing exogenous melatonin into this system restored metabolic parameters to levels observed within control animals. Similarly, in mice fed a high fat diet (HFD), exogenous melatonin administration was sufficient to restore diminished insulin sensitivity and glucose tolerance (5). Consequently, another study demonstrated that daily melatonin administration was sufficient to decrease the bodyweight gain of HFD fed rats by 54% compared to HFD rats not treated with melatonin (6).

**Figure 1:**
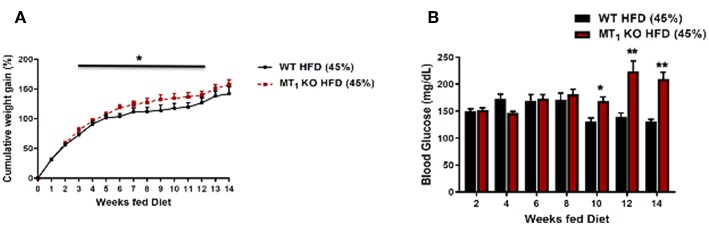
Removal of MT_1_ alters the metabolic response of C3H mice to DIO. Male mice were fed *ad libitum* with a HFD (D12451, 45% kcal/fat, Research Diets Inc.) from weaning at 4 weeks of age until 20 weeks of age. **(A)** MT_1_ KO mice showed a small, but significant cumulative weight gain with respect to control **(B)**. Fasting glucose levels were significantly higher in MT_1_ KO mice after 10 weeks of HFD. Data are presented as mean ± SEM (*n* = 8–10); *p < 0.05, **p < 0.01 WT vs. MT_1_ KO Two-Way ANOVA (*post-hoc*: Holm-Sidak).

These data suggest that melatonin may be functioning, at least in part, to alleviate a number of metabolic consequences associated with diet-induced obesity (DIO). To this end, we have recently examined the effects of DIO on body weight, food intake, and related metabolic parameters within WT and MT_1_ KO mice fed a HFD. Our data demonstrate that DIO elicits markedly higher cumulative weight gain and hyperglycemia within MT_1_ KO mice. Collectively, our data highlight that signaling through MT_1_ may in fact regulate a number of protective metabolic responses during the course of DIO, thus raising the intriguing possibility that MT_1_ may offer a unique therapeutic target for counteracting metabolic consequences elicited by DIO (Figure 1).

Previous studies have established a potential role for melatonin in the regulation of body fat by demonstrating that melatonin administration leads to a reduction in body fat content (7).

Moreover, correlations exist between aging and the reduction of pineal melatonin production, increased body weight, visceral fat, and high levels of leptin and insulin (7). In 1985, a seminal study by Bartness and Wade (8) demonstrated that in Siberian hamsters, melatonin treatment had the ability to decrease body weight, carcass lipid content, and food intake without affecting spontaneous locomotor activity. Years later, Rasmussen et al. (9) found that middle-aged rats treated with melatonin began to display decreased visceral fat, leptin, and insulin to levels that were found in young animals. In addition, in a crossover study, rats initially treated with melatonin rapidly began to increase body weight when switched to the control treatment, and rats that started as control animals rapidly decreased their body weight in response to melatonin treatment (10).

The progression of obesity is a complex process underlined by chronic inflammation (11). Interestingly, in a genetic mouse model of obesity, melatonin treatment was capable of ameliorating both inflammatory infiltration and obesity-induced adipokine alteration (12). Melatonin treatment has also been shown to reduce fat mass (and percentage of body fat) with a concomitant increase of lean mass in postmenopausal women following 1 year of treatment compared to placebo (13). Studies now suggest that melatonin treatment may stimulate lipolysis through the activation of the sympathetic nervous system, as well as stimulate intramuscular adipocyte lipolysis via activation of both extracellular signal-regulated kinase (ERK) 1/2 and protein kinase A (PKA) signaling (14, 15). Taken together, these studies begin to demonstrate a potential role for melatonin in modulating body weight, specifically through its regulation of fat mass.

To date, few studies have provided insight as to whether melatonin's regulation of body composition involves the modulation of feeding behavior. A recent study highlighted that MT_1_ signaling stimulates the transcription *Pro-opiomelanocortin* (*Pomc*) mRNA in the hypothalamus and pituitary and the removal of this receptor results in mice spending substantially more time feeding (16). In addition, direct melatonin infusion via intracerebroventricular cannulation has been shown to reduce food intake (17), thus providing further evidence that melatonin might play an important role in modulating the circuitry that regulates feeding behavior.

## Interplay Between Melatonin and Leptin

Leptin is an adipose-derived hormone that is released in a circadian manner by adipose tissue. Plasma leptin levels peak late within the dark cycle and subsequently decrease during the light cycle (18, 19). Because of the circadian nature of melatonin's secretion—peaking at night—melatonin has been thought to be a circadian timing cue for leptin secretion. Indeed, melatonin has been shown to drive the daily rhythm of plasma leptin, and when it is absent the leptin rhythm is severely blunted in Syrian hamsters (20). Moreover, Buonfiglio et al. (17) demonstrated that the long-term absence of circulating melatonin leads to impairments in leptin signaling and leptin resistance within the hypothalamus. Obese individuals have high levels of leptin, however due to leptin resistance their regulation of food intake—and consequently body weight regulation—is impaired. Therefore, leptin resistance, which is induced either by pinealectomy or genetic removal of melatonin receptors, increases the expression of a number of orexigenic genes such as *Agouti-related protein* (*Agrp*) and *Neuropeptide Y* (*Npy*), which are modulated by leptin signaling.

These changes result in increased long-term food intake and weight gain. Interestingly, administration of exogenous melatonin prevented the negative effects induced by pinealectomy and reduced the expression of both *Agrp* and *Orexin*, thus leading to reductions in food intake, weight gain, leptin levels, and adipose fat pads (17). Consistent with these findings, Río-Lugos et al. (21) also demonstrated that melatonin treatment was able to decrease high levels of circulating leptin and adiponectin, as well as down-regulate the expression of *Npy*—a strong orexigenic signal—that was observed in HFD fed rats.

Finally, a recent study reported that MT_1_ KO mice are leptin resistant compared to controls since the administration of leptin failed to induce signal transducers and activators of transcription 3 (STAT3) phosphorylation in the arcuate nucleus (22). Consistent with these results, leptin receptor mRNA levels in the hypothalamus of MT_1_ KO were reduced (about 50%) with respect to mRNA levels in controls (22). Thus, the lack of MT_1_ signaling induces leptin resistance by down-regulation of the leptin receptor.

## Melatonin and Energy Expenditure

In addition to the modulatory role of melatonin on energy intake, it seems that melatonin may also play an important role in modulating energy expenditure. In an experimental model of obesity and type 2 diabetes mellitus, using Zücker diabetic fatty rats, melatonin treatment induced browning of inguinal white adipose tissue (WAT) and increased Brown adipose tissue (BAT) weight with thermogenic properties (23, 24). Buonfiglio et al. (17) has demonstrated that pinealectomy decreased the amount of uncoupling protein 1 (UCP1) in BAT, thereby indicating lower thermogenic activity after cold exposure (25). Tan et al. (26) suggested that there is a correlation between light exposure at night and bodyweight gain in humans, and if melatonin recruits BAT in humans—as it does in other species—individuals who have their endogenous melatonin decreased by experiencing long daily photoperiods should have less functional BAT and may gain more bodyweight.

Recent studies suggest that gut microbiota composition is also correlated with metabolic disorders and obesity (27, 28). Surprisingly, it was demonstrated that melatonin treatment following DIO in mice, was capable of modulating gut microbiota back to levels observed in lean mice, and provides beneficial effects against obesity, insulin resistance, liver steatosis, and low-grade inflammation in HFD-fed mice (29). Although there remains much that still needs to be understood regarding the role of melatonin in energy homeostasis, there is significant data in different species that now demonstrates that melatonin has an anti-obesogenic effect and that it is involved in the regulation of all three of the main steps of energy balance: energy intake, energy storage, and energy expenditure.

## Melatonin Receptors Involvement in the Regulation of Glucose Metabolism

**Figure 2:**
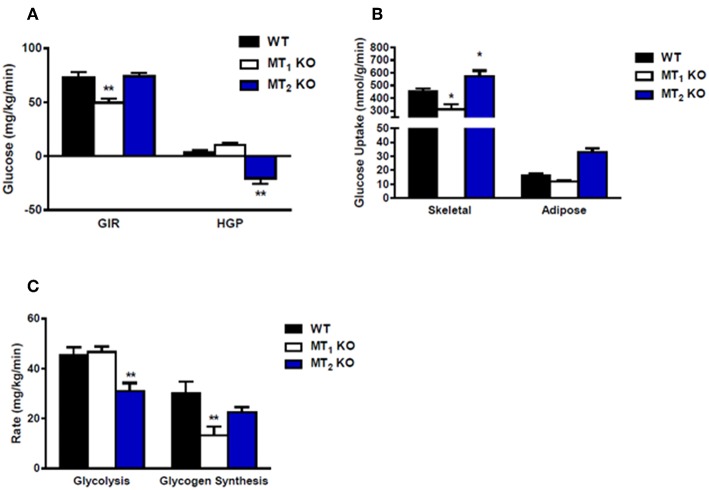
Loss of MT_2_ does not induce systemic insulin resistance in male mice. **(A)** Hyperinsulinemic-euglycemic clamp in awake mice. Glucose infusion rate (GIR) was not different between WT and MT_2_ KO mice, whereas hepatic glucose production (HGP) was significantly lower in MT_2_ KO mice. However, since negative HGP rates are not physiologically possible, the values observed in MT_2_ KO mice likely arise from a slight underestimation of glucose disposal rates. **(B)** Insulin-stimulated glucose uptake in skeletal muscle (gastrocnemius) and white adipose tissue (epididymal) is higher in MT_2_ KO with respect to WT and MT_1_ KO. **(C)** Whole body glycolysis and glycogen synthesis. Glycolysis was lower in MT_2_ KO mice while glycogen synthesis was not different between MT_2_ KO and WT. Taken together these data suggest that while male MT_1_KO mice show systemic insulin resistance with respect to WT, MT_2_ KO do not exhibit insulin resistance. Results are expressed as mean ± SEM (*n* = 5–7 WT, MT_1_ and *n* = 12 MT_2_; Two-way ANOVA *P < 0.05; **P < 0.01, *post-hoc*: Holm-Sidak). Hyperinsulinemic-euglycemic clamp was performed as described in Owino et al. (30).

Collectively, much of the work obtained using mice lacking melatonin receptors has established a generally beneficial role for melatonin on glucose metabolism. Recent studies using these models have demonstrated that in the mouse genetic ablation of MT_1_ or MT_2_ affects glucose metabolism. MT_1_ KO mice display systemic insulin resistance marked by impaired skeletal muscle glucose uptake, adipose tissue glucose uptake, and significantly reduced liver insulin sensitivity (30). This effect appears to involve modulation of phosphatidylinositol-3-kinase (PI3K)-protein kinase B (AKT) activity and fits nicely with previous work demonstrating the ability of melatonin to inhibit hepatic gluconeogenesis (31), and stimulate glucose uptake within both skeletal muscle cells (32) and primary adipocytes (33). MT_2_ KO mice, which previously lacked a clear metabolic phenotype, were recently reported to display decreased hepatic insulin sensitivity and increased insulin secretion (34). These findings differ quite drastically from a previous report demonstrating that MT_2_ KO male mice are neither insulin resistant nor glucose intolerant (35) (see also Figure 2). The discrepancy between these findings may lie in the fact that the earlier metabolic characterization of MT_2_ KO mice utilized male mice (35) whereas the more recent study was performed utilizing female mice (35). Sex differences in insulin sensitivity have been reported for both humans and rodents (36, 37), and as such, the divergence in these findings raises the intriguing possibility that melatonin receptors may potentially be regulated differently between males and females. Additional studies need to be done to confirm that gender is in fact a variable responsible for the observed differences in insulin sensitivity within MT_2_ KO mice.

Thus far, efforts in understanding the role of melatonin on glucose metabolism have been focused heavily on pancreatic islets (38). Although valuable, this focus somewhat overshadows the effects of melatonin on other tissues (skeletal muscle, adipose tissue, liver), as well as its effects on the brain, where both melatonin receptor subtypes are highly expressed within the SCN (2). In the SCN, MT_1_, and MT_2_ function intricately together to entrain and synchronize the rhythm of the master circadian clock (39–41). It is now recognized that disruption of the circadian clock is linked to a number of metabolic disorders (42), and as such, the contribution of melatonin to clock entrainment and its potential implications on metabolism should not be overlooked in the design of future studies. The disruption of independent peripheral tissue clocks within insulin sensitive tissues has also been linked to a number of metabolic disorders (43–46). However, to date, current studies do not lend strong support to the notion that melatonin directly regulates metabolic parameters through its regulation of peripheral tissue clocks. Examination of the effect of melatonin receptor removal on clock genes within the pancreas, adipose tissue, and liver demonstrate only marginal effects on clock gene expression, amplitude and phase (47, 48).

Since the majority of the studies discussed above are performed in rodent models, another important point that must be considered in the interpretation of these results is that rodents are nocturnal and undergo a peak in melatonin synthesis during their active phase, whereas humans are diurnal and experience peak melatonin levels during their inactive “sleep phase.” This difference suggests there may exist different functional requirements for both direct and indirect effects of melatonin between rodents and humans. Interestingly, a number of studies now highlight the importance of “timing” and the “delayed effects” of melatonin in interpreting results from melatonin signaling studies. In the case of insulin signaling, this was eloquently shown in a recent study highlighting the ability of nocturnal activation of MT_1_ to modulate insulin sensitivity during the day (30). Moving forward in attempts to reconcile data collected from human and rodent models, it will be important to keep in mind potential differences that may exist pertaining to both the timing and functional constraints of melatonin in each species.

It is worth noting that a series of recent studies have reported that melatonin synthesis occurs in the mitochondria of neurons where MT_1_ receptors are also present (49). Since mitochondria are key regulators of cellular metabolism, it would be interesting to see how dysfunction of melatonin synthesis and signaling within the mitochondria may contribute to the regulation of energy metabolism and expenditure.

## Role of Melatonin Receptors Variants in Human Metabolic Disorders

Over the years, genetic variants in melatonin receptors have been associated with a number of metabolic disorders such as type 2 diabetes (T2D), gestational diabetes mellitus (GDM) and obesity (50–55). Recently, there has been considerable excitement directed toward the role of melatonin in the regulation of T2D. Much of this interest has come from a series of recent genomic-wide association studies (GWAS) linking *MTNR1B*, the genetic locus encoding MT_2_, to increased fasting blood glucose levels and T2D risk (56, 57). An early study examined the association of six synonymous *MTNR1B* variants (G24E, L60R, V124I, R138C, R231H, and K243R) with obesity and T2D in a Danish and French population (57). None those variants were associated with T2D; however, G42E (rs8192552) was associated with decreased fasting plasma glucose. Furthermore, rs8192552 was also found to be associated with an increased prevalence of obesity, increased body mass index (BMI), and waist circumference.

In total, forty synonymous *MTNR1B* variants were later found to be associated with T2D. From these forty variants, four complete loss of function variants (A24P, L60R, P95L, and Y308S) – which lost the ability to bind melatonin and activate downstream Gi dependent signaling—were uncovered (58). In 2018, the same group under the lead of Karamitri reported their assessment of all forty variants on multiple pathways thought to be regulated by *MTNR1B* activation (58). They found that in addition to the four loss of function variants (A24P, L60R, P95L, and Y308S), four additional variants (S123R, R138C/H/L, F250V, and R316H) remarkably impaired cAMP responses (58).

A study from the DIAGRAM (DIAbetes Genetics Replication and Meta-analysis) consortium further aimed to identify a credible set of variants that overlap with FOXA2 binding sites as a causal mechanism for T2D susceptibility. Interestingly, *MTNR1B* variant rs10830963 was implicated as driving T2D association (59). A subsequent group did not manage to identify any association of rs10830963 and rs1387153 with T2D and fasting blood glucose (FBG) in their Indian population (60). However, when their data was subsequently stratified according to BMI, rs1387153 showed a strong association with low FBG levels in low BMI groups. Similar results were reported by Gan et al. in a Chinese population where rs10830963 didn't show an association with T2D (61). Although not associated with T2D, rs10830963 was found to be associated with GDM in a European cohort (62).

Collectively, genetic studies highlighting the association of *MTNR1B* to T2D risk have reconciled on the ability of two frequent variants (SNPs rs1387153 and rs10830963) to modulate insulin secretion from pancreatic beta cells (55, 57, 62–65). Interestingly, carriers of the rs10830963 risk allele express substantially higher levels of *MTNR1B* mRNA within their pancreatic islets when compared to non-carriers (57). This observation has fueled the general notion that increased melatonin signaling—as a consequence of increased receptor expression levels—likely inhibits pancreatic insulin secretion and increases T2D risk. These results suggest an overall *negative* effect of melatonin on diabetes progression; however, it is important to note that increased *MTNR1B* expression at the mRNA level may not correlate to increased receptor protein levels and/or receptor signaling within these islets. Moreover, these findings appear to be in conflict with a recent clinical study demonstrating that decreased nocturnal melatonin levels are in fact associated with increased, not decreased, risk for diabetes (66).

Although *MTNR1A* variants have not been directly associated with T2D, a few studies have reported an association of *MTNR1A* variants with polycystic ovary syndrome (PCOS). Patients affected by PCOS often develop insulin resistance and T2D. Finally, it is worth mentioning that a reduction in *MTNR1A* mRNAs has been observed in the liver of T2D patients that were unable to control glucose levels (30). Thus, experimental evidence also supports a possible role for MT_1_ in T2D (30, 53, 67).

## Conclusions and Future Perspective

The experimental evidence accumulated thus far indicates that melatonin plays an important role in the regulation of glucose and metabolism are quite solid with a substantial amount of work conducted in human studies. Nonetheless, there continues to be a need for more conclusive studies to fully elucidate the exact role which melatonin and its associated receptors contribute to the regulation of different metabolic processes within the body.

## Author Contributions

SO, DB, CT, and GT wrote the paper. SO drew the figures.

### Conflict of Interest Statement

The authors declare that the research was conducted in the absence of any commercial or financial relationships that could be construed as a potential conflict of interest.
